# Supplementary material for: A Critical View of the Application of the APEX Software (Aqueous Photochemistry of Environmentally-Occurring Xenobiotics) to Predict Photoreaction Kinetics in Surface Freshwaters
Source: Molecules. 2019 Dec 18;25(1):9. doi: 10.3390/molecules25010009 (PMC7017383; doi:10.3390/molecules25010009)
Supplement: Supplementary file 1 [file molecules-25-00009-s001.zip › 3.2.4_gcc-4.4.0.zip!/3.2.4_gcc-4.4.0/3.2.4_gcc-4.4.0/doc/octave/html/interpreter/File-Archiving-Utilities.html]

File Archiving Utilities - Untitled


Next: Networking Utilities,
Previous: Filesystem Utilities,
Up: System Utilities


---

### 34.3 File Archiving Utilities

— Function File: **bunzip2** (bzfile, dir)  
> Unpack the bzip2 archive bzfile to the directory dir. If
> dir is not specified, it defaults to the current directory.
>
>
>
> **See also:** unpack, bzip2, tar, untar, gzip, gunzip, zip, unzip.

— Function File: entries = **gzip** (files)  
— Function File: entries = **gzip** (files, outdir)  
> Compress the list of files and/or directories specified in files.
> Each file is compressed separately and a new file with a '.gz' extension
> is created. The original files are not touched. Existing compressed
> files are silently overwritten. If outdir is defined the compressed
> versions of the files are placed in this directory.
>
>
>
> **See also:** gunzip, bzip2, zip, tar.

— Function File: **gunzip** (gzfile, dir)  
> Unpack the gzip archive gzfile to the directory dir. If
> dir is not specified, it defaults to the current directory. If
> the gzfile is a directory, all files in the directory will be
> recursively gunzipped.
>
>
>
> **See also:** unpack, bunzip2, tar, untar, gzip, gunzip, zip, unzip.

— Function File: entries = **tar** (tarfile, files, root)  
> Pack files files into the TAR archive tarfile. The
> list of files must be a string or a cell array of strings.
>
> The optional argument root changes the relative path of files
> from the current directory.
>
> If an output argument is requested the entries in the archive are
> returned in a cell array.
>
>
>
> **See also:** untar, gzip, gunzip, zip, unzip.

— Function File: **untar** (tarfile, dir)  
> Unpack the TAR archive tarfile to the directory dir.
> If dir is not specified, it defaults to the current directory.
>
>
>
> **See also:** unpack, bunzip2, tar, gzip, gunzip, zip, unzip.

— Function File: entries = **zip** (zipfile, files)  
— Function File: entries = **zip** (zipfile, files, rootdir)  
> Compress the list of files and/or directories specified in files
> into the archive zipfiles in the same directory. If rootdir
> is defined the files is located relative to rootdir rather
> than the current directory
>
>
>
> **See also:** unzip, tar.

— Function File: **unzip** (zipfile, dir)  
> Unpack the ZIP archive zipfile to the directory dir.
> If dir is not specified, it defaults to the current directory.
>
>
>
> **See also:** unpack, bunzip2, tar, untar, gzip, gunzip, zip.

— Function File: **pack** ()  
> This function is provided for compatibility with matlab, but it
> doesn't actually do anything.

— Function File: files = **unpack** (file, dir)  
— Function File: files = **unpack** (file, dir, filetype)  
> Unpack the archive file based on its extension to the directory
> dir. If file is a cellstr, then all files will be
> handled individually. If dir is not specified, it defaults to
> the current directory. It returns a list of files
> unpacked. If a directory is in the file list, then the
> filetype to unpack must also be specified.
>
> The files includes the entire path to the output files.
>
>
>
> **See also:** bunzip2, tar, untar, gzip, gunzip, zip, unzip.

— Function File: entries = **bzip2** (files)  
— Function File: entries = **bzip2** (files, outdir)  
> Compress the list of files specified in files.
> Each file is compressed separately and a new file with a '.bz2' extension
> is created. The original files are not touched. Existing compressed files
> are silently overwritten.If outdir is defined the compressed versions
> of the files are placed in this directory.
>
>
>
> **See also:** bunzip2, gzip, zip, tar.
